# Multimorbidity Through the Lens of the Eye: Pathogenic Variants for Multiple Systemic Disorders Found in an Autosomal Dominant Congenital Cataract Cohort

**DOI:** 10.3390/genes16050604

**Published:** 2025-05-20

**Authors:** Vanita Berry, Manav B. Ponnekanti, Nancy Aychoua, Alex Ionides, Chrysanthi Tsika, Roy A. Quinlan, Michel Michaelides

**Affiliations:** 1 UCL Institute of Ophthalmology, University College London, 11-43 Bath Street, London EC1V 9EL, UK; nancy.aychoua@nhs.net; 2 Moorfields Eye Hospital NHS Foundation Trust, London EC1V 2PD, UK; a.ionides@nhs.net (A.I.); c.tsika@ucl.ac.uk (C.T.); 3 UCL Medical School, University College London, 74 Huntley St, London WC1E 6DE, UK; m.ponnekanti@ucl.ac.uk; 4 Department of Biosciences, University of Durham, Durham DH1 3LE, UK; r.a.quinlan@durham.ac.uk

**Keywords:** autosomal dominant congenital cataract, multimorbidity, WES

## Abstract

Background: This paper will identify the potential genetic causes of multimorbidity associated with autosomal dominant congenital cataract (ADCC). Methods: Whole exome sequencing (WES) was performed on 13 individuals affected with ADCC. Subsequent bioinformatic analyses identified variants with deleterious pathogenicity scores. Results: Disease-causing variants were identified in 8 genes already linked to cataract (*CHMP4B*, *CRYAA*, *CRYBA1*, *CRYGD*, *CYP21A2*, *GJA8*, *OPA1*, and *POMGNT1*), but variants previously associated with systemic disorders were also found in a further 11 genes (*ACTL9*, *ALDH18A1*, *CBS*, *COL4A3*, *GALT*, *LRP5*, *NOD2*, *PCK2*, *POMT2*, *RSPH4A*, and *SMO*). All variants were identified via pipeline data analysis, prioritising rare coding variants using Kaviar and the Genome Aggregation Database. The following ADCC-associated non-ocular phenotypes were identified in four patients in the cohort: (i) Horner’s pupils, vaso-vagal syncope, and paroxysmal orthostatic tachycardia syndrome; (ii) reduced kidney function and high cholesterol; (iii) hypertension, high cholesterol, and kidney stones; and (iv) grade 1 spondylolysis. Conclusions: We report 11 novel genes identified in an ADCC patient cohort associated with systemic disorders found, along with 8 known cataract-causing genes. Our findings broaden the spectrum of potentially cataract-associated genes and their related lens phenotypes, as well as evidence multimorbidities in four patients, highlighting the importance of careful multisystem phenotyping following genetic analysis.

## 1. Introduction

The advent of whole exome/genome sequencing has revealed patterns of multimorbidity through defined pipeline data analysis. These patterns would be impossible to establish via traditional linkage mapping, in which the search for linkages is confined to manifested and clinically evident disease phenotypes. Multimorbidity describes the presence of multiple variants which contribute to distinct diseases within the same patient, not necessarily as part of a defined syndrome. Thirty years ago, through conventional methods, we identified disease-causing variants in several novel genes responsible for congenital cataract; the aforementioned methodological advances allow us to better understand the co-variant nature of the pathologies that manifest alongside cataract in these cases.

Cataract, characterised by the clouding of the ocular lens, occurs both independently and as part of a broader spectrum of eye anomalies. Congenital cataract exhibits substantial phenotypic diversity; types include nuclear, lamellar, posterior subcapsular, and cortical [1,2,3]. They are typically identified at birth or within the first ten years of life, predominantly exhibiting autosomal dominant inheritance with more sporadic cases of autosomal recessive and X-linked patterns [4].

To date, over sixty genes linked to congenital cataract have been identified, largely presenting as isolated. Among these are genes that encode soluble lens proteins, such as α-, β-, and γ-crystallins; lens-associated membrane proteins, including connexins, aquaporins, and receptor tyrosine kinase EPH receptor A2; Wolframin, an endoplasmic reticulum-associated membrane protein; chromatin modifier protein-4B; cytoskeletal components, such as BFSP1 (filensin), BFSP2 (CP49, phakinin), and vimentin; and transcriptional or developmental regulators, notably *EYA1*, *MAF*, *FOXE3*, *VSX2*, *PAX6*, *PITX3*, and *HSF4* (https://cat-map.wustl.edu/, accessed on 3 February 2025) [4,5].

Congenital cataract is also associated with nearly 300 syndromes [6,7,8], such as congenital adrenal hyperplasia [9] and Crohn’s disease [10]. Here, we report disease-causing variants in the following 19 genes: *SMO*, *COL4A3*, *ACTL9*, *OPA1*, *RSPH4A*, *PCK2*, *NOD2*, *CBS*, *GALT*, *ALDH18A1*, *CHMP4B*, *LRP5*, *POMGNT1*, *POMT2*, *GJA8*, and *CRYAA*. Eleven of these are potentially linked to multiple systemic disorders found in our ADCC cohort within a British population, serving as an example of potential multimorbidities associated with cataract.

## 2. Materials and Methods

### 2.1. Phenotyping

The patients identified in this ADCC panel were attending the Genetic Service at Moorfields Eye Hospital, London, UK. The study protocol adhered to the Tenets of the Declaration of Helsinki and was approved by the UCL research ethics committee (project ID-4817/001). All the members participating in this study gave written informed consent and underwent full ophthalmic examination, and initially, all affected individuals were diagnosed as having isolated bilateral congenital cataract.

### 2.2. Whole Exome Sequencing (WES) and Bioinformatic Analyses

Genomic DNA was extracted from EDTA-treated blood samples using the Nucleon II DNA Extraction Kit (Scotlab Bioscience, Strathclyde, UK). Exome capture and enrichment were performed with the SureSelectXT Human All Exon V6 kit (Agilent Technologies, Santa Rosa, CA, USA). Sequencing was carried out in paired-end mode on an Illumina HiSeq 2500 platform (Macrogen Europe, Amsterdam, Netherlands), achieving an average coverage depth of 50×. Raw sequencing data in FASTQ format were aligned to the human reference genome (GRCh37/hg19, UCSC Genome Browser) and analysed using the Phenopolis bioinformatics pipeline [11].

Variant prioritisation was conducted using a tier-based approach. Initial filtering selected rare coding variants with an allele frequency < 0.0001 from the Kaviar database (http://db.systemsbiology.net/kaviar/, accessed on 4 March 2021) [12] and the Genome Aggregation Database (gnomAD v2.1; http://gnomad.broadinstitute.org/, accessed on 4 March 2021) [13], specifically within known cataract-associated genes listed in Cat-Map (https://cat-map.wustl.edu/, accessed on 3 February 2025). Variants were then ranked by combined annotation dependent depletion (CADD) scores, prioritising those predicted to be moderately or highly deleterious (CADD > 15). Further bioinformatic validation and the annotation of selected variants were performed using VarSome (version 11.9; https://varsome.com, accessed on 24 June 2024) (Table 1).

### 2.3. Sanger Sequencing

Direct Sanger sequencing was performed to validate the variants identified by whole exome sequencing. Genomic DNA was amplified by PCR using GoTaq 2X master mix (AB gene; Thermo Scientific, Epsom, UK). The specific forward and reverse primers for *SMO*, *COL4A3*, *ACTL9*, *OPA1*, *RSPH4A*, *PCK2*, *NOD2*, *CBS*, *GALT*, *POMT2*, *ALDH18A1*, *CHMP4B*, *LRP5*, *POMGNTI*, *POMT2*, *CRYAA*, and *GJA8* were designed with https://bioinfo.ut.ee/primer3-0.4.0/, accessed on 16 August 2024; please note that all *GJA8* variants were designed from one set of primers (Table 2). PCR conditions were as follows: 94 °C for 5 min of initial denaturation followed by 30 cycles of amplification of 30 s at 94 °C denaturing, 30 s at 60 °C annealing, and 45 s at 72 °C for extending. After cleaning, the PCR products were reacted with BigDye Terminator v3.1; they were run on ABI 3730 Genetic Analyzer (both from Applied Biosystems, Foster City, CA, USA) and analysed using SeqMan Pro (version 8.0.2 from DNASTAR) sequence analysis. The variants were validated in all affected and unaffected individuals.

## 3. Results

DNA samples from the affected individuals were sent for WES. These patients were clinically examined for isolated congenital cataract, but next generation sequencing technology has enabled multiple disease genes to be found in these patients. After the Phenopolis genetic variant analysis pipeline, we found 22 disease-causing variants in 8 known genes causing congenital cataract, along with 11 novel genes linked to other human disorders. All identified variants in this study are heterozygous (Table 3).

Patient 1 had congenital bilateral posterior polar cataract. We filtered the following:

(i) A novel pathogenic mis-sense variant in *SMO* (NM_005631.5: c.1801G>A; p.A601; exon 10; chromosome 7q32.1);
(ii) A known pathogenic mis-sense variant in *COL4A3* (NM_000091.5: c.746C>T; p.T255M; exon 13; chromosome 2q36.3);
(iii) A known pathogenic nonsense variant in *ACTL9* (NM_178525.5: c.1209C>G; p.Y403*; exon 1; chromosome 19p13.2);
(iv) A pathogenic mis-sense variant in *OPA1* (NM_130836.3: c.1257A>G; p.I419M; exon 13; chromosome 3q29).

**Figure 1:**
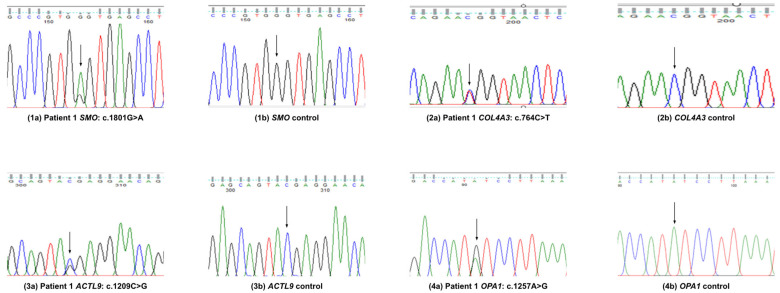
Variants validation by Sanger sequence analysis in patient 1.

After identifying these variants, we re-contacted the patient and obtained further clinical information. This patient also suffers from Horner’s pupil along with vaso-vagal syncope and also has paroxysmal orthostatic tachycardia syndrome (Figure 1).

Patient 2 had bilateral congenital cataract, which was operated on at the age of 18 months. She has one daughter and one son; both are affected with congenital cataract. Her son had left eye aphakic glaucoma and nystagmus. Her daughter had cataract bilateral surgery in infancy but had no glaucoma. Her son also suffers from gallstones, and his gallbladder was removed recently. He has three children, and two of them are affected with congenital cataract. We identified the following:

(i) A novel likely pathogenic variant in *RSPH4A* (NM_001010892.3: c.1129delG; p.E377Kfs*11; exon 3; chromosome 6q22.1);
(ii) A nonsense variant (NM_ 004563.4: c.424C>T; p.R142*) in *PCK2* with uncertain significance;
(iii) A frameshift variant in *NOD2* (NM_022162.3: c.3019dup; p.L1007P*; exon 11; chromosome 16).

**Figure 2:**
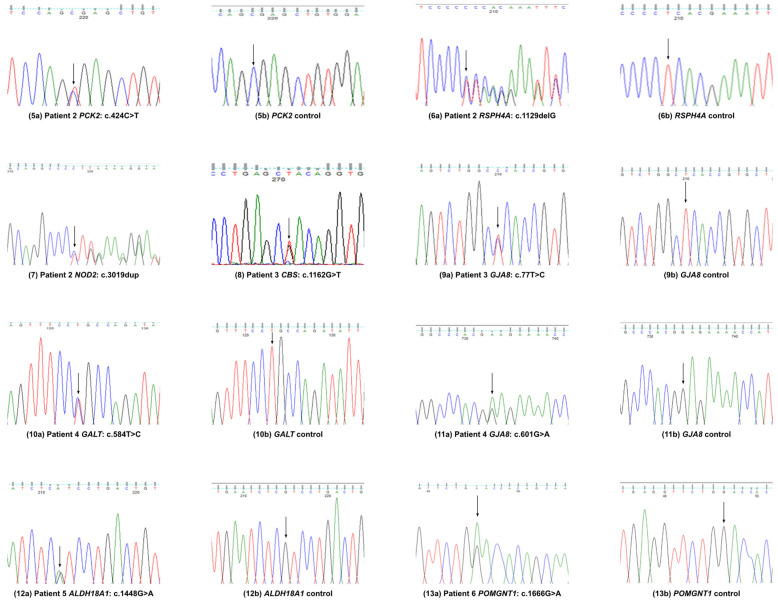
Variants validation by Sanger sequence analysis in patients 2–6.

After finding multiple genes through WES, the proband confirmed that they suffer from gallbladder issues, reduced kidney function, and high cholesterol (Figure 2).

In patient 3, we identified variants in the following three genes:

(i) A novel variant in *GJA8* (NM_005267.5: c.77T>C; p.L26P; exon 2; chromosome 1) causing congenital cataract;
(ii) A novel *CBS* variant (NM_001178008.2: c.1162G>T; p.D388Y; exon 13; chromosome 21).

A known *CYP21A2* variant (NM_000500.9: c.955C>T; p.Q319*, exon 8; chromosome 6) has been previously identified [10]. Clinical correlation for these variants has not been established in this patient, but they could have been responsible for undocumented eye problems or other health issues. No further clinical information was available (Figure 2).

In patient 4, we found the following:

(i) A novel variant in *GJA8* (NM_005267.5: c.601G>A; p.E201K; exon 2; chromosome 1);
(ii) A recurrent heterozygous pathogenic variant in the *GALT* gene (NM_000155.4: c.584T>C; p.L195P; exon 7; chromosome 9p13.3).

This patient also had a *CRYAA* (p.A152del) pathogenic variant, identified previously; no further clinical information was available (Figure 2).

Patient 5 suffered from bilateral congenital cataract and previously had a recurrent pathogenic *CRYAA* (p.R116C) variant. We found that they have another likely pathogenic variant in *ALDH18A1* (NM_002860.4: c.1448G>A; p.R483H; exon 12; chromosome 10q24.1). No further clinical information was available for this patient (Figure 2).

Patient 6 was found to have autosomal dominant bilateral congenital pulverulent cataracts, bilateral aphakia, bilateral retinal detachment, suspected glaucoma, and naevus. Through WES analysis, we found the following:

(i) A pathogenic variant in the *CRYGD* gene (NM_006891.4: c.470G>A; p.W157*; exon 3; chromosome 2q33.3);
(ii) A novel variant in *POMGNT1* (NM_001243766.2: c.1666G>A; p.D556N; exon 20; chromosome1p34.1).

Variants in the latter gene are known to cause cataract and other eye problems. After finding the second gene through WES, we managed to contact the family, and the proband confirmed that they suffer from hypertension, high cholesterol, and kidney stones. Congenital cataract runs in their family; the proband’s daughter was operated on at the age of 6 weeks. The family also has a history of several other eye problems (Figure 2).

Patient 7, who had autosomal dominant nuclear cataract, was identified with the following:

(i) A known variant in *CRYBA1* (NM_005208.5: c.272_274del; p.G91del; exon 4; chromosome 17);
(ii) A novel variant in *CRYAA* (NM_000394.4: c.392G>T; p.C131F; exon 3; chromosome 21q22.3).

**Figure 3:**
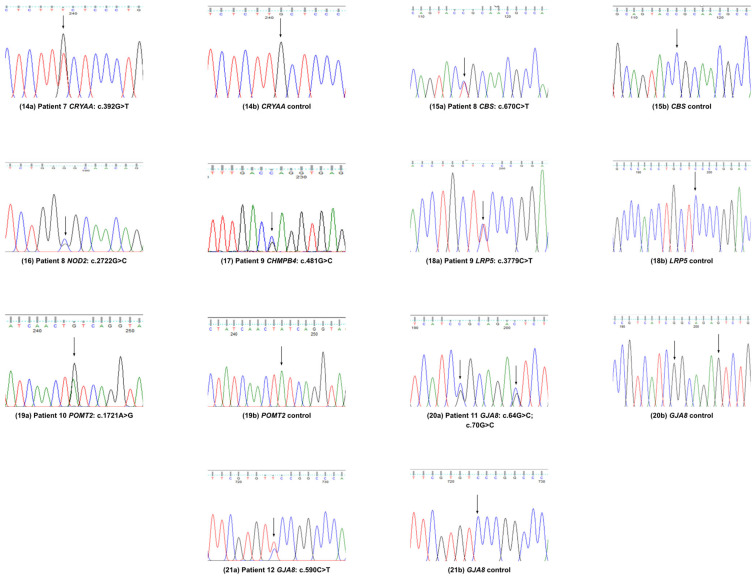
Variants validation by Sanger sequence analysis in patients 7–12.

No further clinical information was available for this patient (Figure 3).

In patient 8, who had congenital cataract, we identified the following:

(i) A mis-sense variant of uncertain significance in *CBS* (NM_000071.3: c.670C>T; p.R224C; exon 8; chromosome 21q22.3);
(ii) A novel mis-sense variant in *NOD2* (NM_022162.3: c.2722G>C; p.G908R; exon 8; chromosome 16) (Figure 3).

It is worth noting that AlphaMissense predicted the *NOD2* (p.G908R) variant to be uncertain/0.45 score, but clinically, its pathogenicity has been validated (https://varsome.com, accessed on 24 June 2024).

Patient 9 had congenital cataract and was operated on at the age of 7 years. We found the following:

(i) A recurrent *CHMP4B* variant (NM_176812.5: c.481G>C; p.E161Q; exon 3; chromosome 20q11.22), known to cause congenital cataract;
(ii) An *LRP5* variant (NM_002335.4: c.3779C>T; p.S1260F; exon 18; chromosome 11) known to cause vitreoretinopathy, primary open-angle glaucoma (POAG), congenital cataract, and osteoporosis–pseudoglioma syndrome (Figure 3).

As soon as a second disease-linked gene was identified, we re-contacted the family, albeit two decades later, and secured additional medical information. Congenital cataract runs in the family. The proband has three sisters, all of whom have CC, high amblyopia, and are blind in one eye. The proband’s three children all have CC and amblyopia. Both the proband and his sisters are around two meters tall, and both suffer from grade 1 spondylolysis.

Patient 10 has a rare heterozygous variant with uncertain significance but high impact in *POMT2* (NM_013382.7: c.1721A>G, p.Y574C; exon 16; chromosome 14). We have no further information for this patient (Figure 3).

Patients 11 and 12 presented with disease-causing variants in *GJA8* causing congenital cataracts. Patient 11 had the following two pathogenic variants:

(i) A recurrent *GJA8* variant (NM_005267.5: c.64G>C; p.G22R);
(ii) A novel *GJA8* variant (c.70G>C; p.G24L; exon 2; chromosome 1q21.1).

Patient 12 had a novel variant in *GJA8* (NM_005267.5: c.590C>T; p.S197F; exon 2; chromosome 1q21.1).

Patient 13 reported with ADCC identified with a recurrent pathogenic variant in *GALT* (NM_000155.4: c.584T>C; p.L195P; exon 7; chromosome 9p13.3), the same as in patient 4. No further clinical information was available for patients 11, 12, and 13 (Table 3), (Figure 3).

## 4. Discussion

All members of the ADCC patient cohort initially presented with isolated congenital cataract. Whole exome sequencing revealed complex genetic architectures, including monogenic, digenic, trigenic, and in one case, tetragenic inheritance patterns, demonstrating significant multimorbidity implications. We report findings from 13 ADCC patients, all of whom are heterozygous in multiple genes, with cataract as the primary clinical phenotype. Variants were identified in the following genes: *SMO*, *COL4A3*, *ACTL9*, *OPA1*, *RSPH4A*, *PCK2*, *NOD2*, *CBS*, *GALT*, *POMT2*, *ALDH18A1*, *CHMP4B*, *LRP5*, *POMGNTI*, *POMT2*, *CRYAA*, *CRYBA1*, *CRYGD*, and *GJA8* (Table 3).

### 4.1. Genes for Congenital Cataract

Out of the 19 genes mentioned above, variants in *GJA8*, *CRYAA*, *CRYBA1*, *CRYGD*, *CHMP4B*, *CYP21A2*, *OPA1*, and *POMGNT1* are primarily responsible for congenital cataract. Here, we discuss the functions of their expressed proteins.

GJA8/Cx50 (gap junction protein alpha 8) encodes a transmembrane connexin protein that is necessary for lens growth and the maturation of lens fibre cells. It is a gap junction component that functions in a calcium and pH-dependent manner. Variants in this gene have been associated with zonular pulverulent cataract, nuclear progressive cataract, and cataract–microcornea syndrome [4]; we found four in our patient panel. The most notable of these are the double pathogenic variants identified in patient 11—a novel p.G22R and a recurrent p.G24L.

CRYAA and CRYBA1 are members of the crystallin family, soluble proteins which are responsible for the development, growth, transparency, and refractive index of the lens. Fifty percent of all non-syndromic inherited congenital cataracts, as well as cataracts associated with other diseases including myopathies, are caused by variants in the crystallin gene family [4]. Here, we have found a novel pathogenic variant in *CRYAA* (p.C131F) in addition to the following previous pathogenic variant found in *CRYBA1* (p.G91del) in patient 7, a demonstration of digenic heterozygosity in a similar manner to patient 11. The loss of the disulphide bridge in CRYAA would be expected to make this chaperone more susceptible to cysteine-directed post-translational modifications, including glutathionylation, which will reduce its chaperone activity [14].

CHMP4B (charged multivesicular body protein 4B) on chromosome 20q is associated with autosomal dominant (posterior polar/subcapsular) cataract [15]. CHMP4B is an integral part of the endosomal sorting complex necessary for transport III membrane modification and scission machinery for networking with GJA8 in the lens [16]. Here, we found a recurrent mis-sense variant in *CHMP4B* (p.E161Q) in patient 9.

CYP21A2 (cytochrome P450 family 21 subfamily A member 2) is associated with congenital adrenal hyperplasia due to 21-hydroxylase deficiency. The cytochrome P450 proteins are monooxygenases, which catalyse many reactions involved in drug metabolism and the synthesis of cholesterol, steroids, and other lipids, playing a significant role in adrenal steroidogenesis [17,18]. In patient 3, earlier, we found a recurrent pathogenic variant in *CYP21A2* (p.Q319* on chromosome 6p21.33) [9].

OPA1 encodes a nuclear-encoded mitochondrial protein similar to dynamin-related GTPases. Heterozygous variants in *OPA1* are a common cause of autosomal dominant optic atrophy as well as Behr syndrome, high myopia, vitreoretinal detachment, and congenital cataracts. Extra-ocular manifestations include sensorimotor neuropathy and ataxia [19]. Recently, compound heterozygous variants in *OPA1* (NM_130837.3: p.S64Nfs*7 and p.I437M) have been reported to have clinical impact in a family [20]. Here, we have identified the same mutation (ENSP00000354681.3, NM_130836.3: c.1257A>G; p.I419M) in patient 1. He suffers from bilateral isolated posterior polar cataract, Adie’s or Horner’s pupil, vaso-vagal syncope, and paroxysmal orthostatic tachycardia syndrome.

POMGNT1 encodes an essential component in the O-mannosylation pathway. Variants in the *POMGNT1* gene in humans cause muscle–eye–brain disease (MEB), which is associated with several ocular abnormalities; retinal dysplasia, ERG abnormalities, and retinal detachments have been reported in patients [21]. *POMGNT1* is also linked to retinitis pigmentosa [22]. Patient 6, who has a variant in this gene, presents with bilateral pulverulent cataracts, bilateral aphakia, bilateral retinal detachment, suspected glaucoma, and naevus. He also suffers from hypertension, high cholesterol, and kidney stones. This patient harboured a recurrent heterozygous variant (c.1666G>A;p.D556N) in *POMGNT1*. A homozygous variant (c.1666G>A; p.D556N) has been reported to cause severe myopia and muscular dystrophies [23].

### 4.2. Genes Associated with Other Systemic Disorders

The remainder of the 19 genes identified in our cohort (*SMO*, *COL4A3*, *ACTL9*, *RSPH4A*, *PCK2*, *NOD2*, *CBS*, *GALT*, *ALDH18A1*, *LRP5*, *POMT2*) are associated with various systemic disorders (Table 3).

SMO encodes a G protein-coupled receptor that functions in hedgehog signal transduction, an essential step during eye development in utero. It is also associated with Curry–Jones syndrome (CJS), a multisystem disorder characterized by patchy skin lesions, polysyndactyly, diverse cerebral malformations, unicoronal craniosynostosis, intestinal malrotation with myofibromas or hamartomas, iris colobomas, and microphthalmia [24]. Twigg et al. reported a recurrent somatic mosaicism for a nonsynonymous variant in *SMO* (c.1234C>T; p.L412F) causing CJS [25]. Other compound heterozygous variants (c.338G>A, p.R113Q, and c.1619C>T, p.A540V) were reported in a patient with both anterior segment dysgenesis (congenital corneal opacity, cataract) and morning glory syndrome [26]. Here, we report a novel pathogenic variant *SMO* (p.A601T) in patient 1 that could potentially contribute to their congenital cataract phenotype.

COL4A3 is responsible for the manufacture of type IV collagen, a multimeric protein composed of 3 alpha subunits, which is the major structural component of basement membranes. COL4A3 is linked to an autosomal recessive form of Alport syndrome, characterized by kidney disease, sensorineural hearing loss, and sometimes, eye abnormalities, such as cataract [27,28]. Here, we report a recurrent pathogenic variant in *COL4A3* (p.T255M) in patient 1, who has congenital cataract. This variant has been reported in Filipino patients with hearing problems [29] and in familial kidney disease [30] without hearing impairment. Recently, Belamkaret et al. (2024) demonstrated that *COL4A3* KO mice exhibit a differential inflammatory and profibrotic response in the cornea, retina, and lens, elaborating the gene’s role in the ocular pathology of Alport syndrome [31].

ACTL9 (actin like 9) is associated with spermatogenic failure 53 and non-syndromic male infertility. Here, we report a nonsense heterozygous variant in *ACTL9* (p.Y403*) in patient 1. As *ACTL9* has not yet been associated with ocular diseases, here, it is likely to be implicated in a systemic disorder. Recently, a homozygous nonsense variant (c.1209C>G; p.Y403*) was reported in a family of Chinese origin as a candidate gene for total fertilization failure [32].

Pathogenic variants in *RSPH4A* (radial spoke head component 4A) are associated with primary ciliary dyskinesia (PCD), a rare genetic ciliopathy. This causes chronic oto-sino-pulmonary infections, with several downstream co-morbidities. This association is well-established; more than 30 pathogenic *RSPH4A* genetic variants have been associated with PCD [33]. Here, we have found a novel likely pathogenic variant with high impact (p.E377Kfs*11) in patient 2. RSPH4A’s role is cataractogenesis can potentially be attributed to the necessity of primary cilia- and centrosome-associated proteins, like CLIC5, for lens development [34].

PCK2 encodes the gluconeogenic enzyme phosphoenolpyruvate carboxykinase 2. Diseases associated with PCK2 include phosphoenolpyruvate carboxykinase deficiency and glycogen storage diseases, the latter of which has been associated with lens opacity [35]. Further, a heterozygous mis-sense variant (c.977C>T; p.P326L) in *PCK2* was found in a Chinese family, causing primary angle-closure glaucoma [36]. Mutations in this gene could also have vascular implications; recent studies have shown that PCK2 also plays a non-enzymatic role in proteostasis, and that loss of PCK2 in endothelial cells impaired vessel sprouting [37]. Here, we have found a novel nonsense variant in *PCK2* (p.R142*) in patient 2.

NOD2 (nucleotide binding oligomerization domain containing 2) plays a significant role in the mediation of innate immunity [38]. Variants in *NOD2* have been associated with Crohn’s disease and Blau syndrome. The specific variant (*NOD2*p.Leu1007fsX1008) found in patient 2 has also been reported to be a strong predictor of the clinical course of Crohn’s disease [39].

CBS (cystathionine beta-synthase) is a key enzyme in the trans-sulphuration pathway; a deficiency of it is associated with homocystinuria. Patients with this condition are frequently affected with ectopic lentis, myopia, retinal detachment, optical atrophy, glaucoma, corneal abnormalities, and cataract [40,41]. It has been suggested that CBS may be a new class of oxidation defence enzyme in eye tissues and in particular in those segments of the eye where constant environmental oxidative stress is imposed [42]. We have found two pathogenic variants in *CBS*: p.D388Y in patient 3 and p.R224C in patient 8.

GALT (galactose-1-phosphate uridyl transferase) plays a key role in galactose metabolism. Variants result in classic galactosemia and can be fatal in newborns. Here, we have found a recurrent mis-sense variant in *GALT* (p.L195P) in patients 4 and 13; this variant accounts for approximately 2.6% of classic galactosemia alleles [43].

ALDH18A1 (aldehyde dehydrogenase 18 family member A1) is involved in the de novo bio-synthesis of ornithine and proline [44]. Variants in this gene are associated with Warburg micro syndrome, which includes intellectual disabilities, muscle weakness, microphthalmia, microcornea, optic atrophy, and cataracts as pathogenic phenotypes. An example of ocular defects associated with gene can be found in Wolthuis et al., who reported a novel, homozygous nonsense variant in *ALDH18A1* (p.Y780C) causing retinopathy along with cutis laxa and fat pads [45]. In patient 5, we have found a variant at p.R483H as well as a recurrent *CRYAA* (p.R116C) variant known to cause ADCC. We have no additional clinical information for this patient.

LRP5 encodes low-density lipoprotein receptor-related protein 5, which is necessary for bone and eye development; dysfunction in this gene is linked to osteoporosis–pseudoglioma syndrome and familial exudative vitreoretinopathy (FEVR) [46,47,48]. Patient 9 has a novel pathogenic variant in *LRP5* (p.S1260F), as well as in *CHMPB4* (p.E161Q). His family members suffer from high amblyopia, congenital cataract, and grade 1 spondylolysis.

POMT2 encodes protein O-mannosyltransferase 2, which is required for interaction with the product of the POMT1 gene for enzymatic function. Defects in this gene are associated with Walker–Warburg syndrome (WWS), an autosomal recessive condition characterised by congenital muscular dystrophy, structural brain defects, and eye malformations [49]. In patient 10, we identified a novel variant in *POMT2* (p.Y574C).

In retrospect, it could be that, in many of these cases, the initial cataract itself is multigenic in cause, with contributions from other disease processes; for example, *CYP21A2*’s role in adrenal steroidogenesis quite possibly has a predisposing effect on lens opacification. However, the precise mechanisms underlying these potential associations remain speculative. To conclusively establish causative relationships, extensive functional studies—including in vitro assays, animal models, or cellular analyses—are essential to elucidate the biological impact of identified genetic variants on lens transparency and cataractogenesis. Addressing this limitation through targeted functional validation of this nature will significantly enhance our understanding of the genetic and molecular underpinnings of cataract formation.

## 5. Conclusions

We highlight potential multimorbidity associated with 22 disease-causing variants across 19 genes identified in patients diagnosed with isolated congenital cataract several decades prior. Our investigation not only expands the mutational spectrum of ADCC but substantiates clinical diagnoses involving multiple body systems with linked co-morbidities.

The variants identified in this study provide compelling evidence of phenotypic heterogeneity, underscoring the importance of correlating clinical observations with next-generation sequencing data. The precise assessment of gene variants is imperative to prevent the misattribution of pathogenicity, with rigorous genotype–phenotype correlations necessary for both predicted pathogenic and benign variants [50]. Such data are necessary to decipher the biological basis of phenotypic variation in familial cataract, serving as a valuable paradigm for understanding the genetic underpinnings of human disease writ large.

Future research involving functional validation via in vitro assays or animal models would further strengthen the utility of our findings. This would facilitate increased diagnostic accuracy, personalised therapeutic approaches, and the development of novel molecular interventions to prevent or delay disease onset. In particular, the early identification of pathogenic variants in children enables proactive clinical surveillance, the timely initiation of targeted therapies, and, wherever possible, preventative interventions, shifting management from reactive to anticipatory care.

## Figures and Tables

**Table 1:** Pathogenicity scores of variants in the following genes.

Genes	HGVSc	HGVSp	GERP	CADD	REVEL	AlphaMissense	In Silico Pathogenicity
*SMO*	c.1801G>A	p.A601T	5.8	28.1	Uncertain /0.53	Benign Supporting/0.24	Novel/Likely Pathogenic
*COL4A3*	c.764C>T	p.T255M	5.6	27.1	Benign Moderate/ 0.30	Benign Moderate/0.09	Likely Pathogenic
*ACTL9*	c.1209C>G	p.Y403*	4.5	36.0	-	-	VUS (ClinVar-Pathogenic)
*OPA1*	c.1257A>G	p.I419M	5.7	24.4	Pathogenic Moderate /0.94	Uncertain/0.55	Recurrent/Likely Pathogenic
*RSPH4A*	c.1129delG	p.E377Kfs*11	5.3	26.1	-	-	Likely Pathogenic
*PCK2*	c.424C>T	p.R142*	5.40	26.1	-	-	VUS
*NOD2*	c.3019dup	p. L1007P*	5.5	35.0	-	-	VUS
*CBS*	c.1162G>T	p.D388Y	4.7	32.0	Pathogenic Moderate /0.87	Uncertain/0.55	Novel/Likely Pathogenic
*GJA8*	c.77T>C	p.L26P	5.0	25.5	Pathogenic/Strong/0.99	Pathogenic/Strong/0.99	Novel/Likely Pathogenic
*GALT*	c.584T>C	p. L195P	4.78	26.1	Pathogenic Moderate /0.92	Uncertain	Recurrent/Pathogenic
*GJA8*	c.601G>A	p.E201K	4.8	27.0	Pathogenic/Strong/0.99	Pathogenic/Strong/0.99	Novel/Pathogenic
*ALDH18A1*	c.1448G>A	p.R483H	5.5	32.0	Pathogenic Moderate /0.85	Pathogenic/Moderate/0.98	Likely Pathogenic
*POMGNT1*	c.1666G>A	p.D556N	6.0	27.0	Uncertain /0.48	Benign Moderate/0.13	Conflicting
*CRYAA*	c.392G>T	p.C131F	3.7	25.0	-	Pathogenic/Moderate/0.97	Novel/Likely Pathogenic
*CBS*	c.670C>T	p.R224C	4.6	27.5	Pathogenic Moderate /0.93	Benign Supporting/0.21	Novel Pathogenic/Moderate
*NOD2*	c.2722G>C	p. G908R	5.9	31.0	-	Pathogenic Supporting/0.84	VUS
*CHMP4B*	c.481G>C	E161Q	6.0	32.0	Uncertain /0.63	Pathogenic Supporting/0.87	Recurrent/Pathogenic
*LRP5*	c.3779C>T	p.S1260F	4.3	26.2	Pathogenic Moderate /0.87	Uncertain/0.6	Pathogenic/Strong
*POMT2*	c.1721A>G	p.Y574C	5.67	26.3	Pathogenic Supporting /0.76	Benign Supporting/0.26	VUS
*GJA8*	c.64G>C	p.G22R	5.03	29	Pathogenic/Strong/0.98	Pathogenic/Moderate/0.99	Recurrent/Pathogenic
*GJA8*	c.70G>C	p.V24L	5.03	-	Pathogenic Moderate /0.86	Pathogenic Supporting/0.78	Novel/Pathogenic
*GJA8*	c.590C>T	S197F	4.8	26	Pathogenic/Strong/0.98	Pathogenic/Moderate/0.99	Novel/Pathogenic

Genomic evolutionary rate profiling (GERP) NR corresponds to the neutral rate conservation score of the site; combined annotation dependent depletion (CADD) is a score for the deleteriousness of a variant. A CADD score > 15 is considered damaging; rare exome variant ensemble (REVEL) is a score to predict the pathogenicity of mis-sense variants based on a combination of scores from 13 individual tools and ranges between 0 to 1, with a higher value indicating that the variant is likely to be deleterious; AlphaMissense predicts the average pathogenicity over all possible mis-sense variants in a gene. * Indicates the truncated protein.

**Table 2:** Primers.

Gene	Forward Primers	Reverse Primers
*SMO*	ttcttcacgctccttcccta	cagaaatatcctggggcagt
*COL4A3*	tttacttacgggccaagctg	aaggacgggaaggaatcaat
*ACTL9*	gcaaggagctgttccagtgt	catggggaaggtgggttta
*OPA1*	gggttgcaattcatttcagtg	gagccatgcctgatgtcac
*RSPH4A*	actgcacccagccaattt	tgcaataacaatttgtgcaggta
*PCK2*	aaagtgggtctagggacaagg	catgctgaatggaagcacat
*NOD2*	tgcaggtacttaaccactatcct	tcagatccttcacatgcaga
*CBS*	accagtgaggtccaggagag	gggggatcaggataaggaca
*GJA8*	tctgcacaaaggaagcactg	gacacagaggccacagacaa
*GALT*	gggtttcttggctgagtctg	tgctaaggcctcctagcaagt
*ALDH18A1*	cctgccaggtctgctacttt	cgttgtgcacatgtaccctaga
*POMGNT1*	gtgggacacacccatgaagt	ttgaagattccagagcaaagg
*CRYAA*	caggggcatgaatccataaa	gggaagcaaaggaagacaga
*CBS*	aaatccccaattctcacatcc	aggagttcaccaaggagagg
*NOD2*	ttgggttaagtttggccatc	ggacaagggacatttccaag
*CHMP4B*	acccctcacagggagtcatt	aagggtcctgatgaatgtgc
*LRP5*	ttctcccagcctctcttctg	cttgttgggcctaaaagaca
*POMT2*	ttatgggagatggaggcttg	catgctgaatggaagcacat

https://bioinfo.ut.ee/primer3-0.4.0/, accessed on 16 August 2024.

**Table 3:** ADCC patients with associated diseases.

Patient	ADCC and Other Identified Pathologies	Gene (Variant)	Inheritance	Pathologies Associated with Gene in the Literature
1.	Posterior polar cataract, Horner’s pupils, vaso-vagal syncope, and paroxysmal orthostatic tachycardia syndrome.	***SMO*** (p.A601T)	AR	Curry–Jones syndrome
				Anterior segment dysgenesis
				Morning glory disc anomaly
				Congenital cataract
		***COL4A3*** (p.T255M)	AD/AR	Alport syndrome (including haematuria)
				Eye abnormalities
				Hearing loss
				Kidney failure
		***ACTL9*** (p.Y403*)	AR	Fertilization failure
		*OPA1* (p.I419M)	AD/AR	High myopia
				Vitreoretinal detachment
				Behr syndrome with early-onset optic atrophy
				Sensorimotor neuropathy
				Ataxia
				Congenital cataract
2.	Bilateral congenital cataract, reduced kidney function, and high cholesterol.	***RSPH4A*** (p.E377Kfs*11)	AD/AR	Primary ciliary dyskinesia
		***PCK2*** (p.R142*)	AR	Primary angle-closure glaucoma
		***NOD2*** (p. L1007P*)	AD/AR	Crohn’s disease
3.	Congenital cataract (no further information)	*CYP21A2* (p.Q319*)	AR	Congenital adrenal hyperplasia
				Congenital cataract
		***CBS*** (p.D388Y)	AR	Homocystinuria with ocular complications
				Osteoporosis
				Vascular complications
				Intellectual disability
				Congenital cataract
		*GJA8* (p.L26P)	AD/AR	Congenital cataract
4.	Congenital cataract (no further information)	*CRYAA* (p.A152del)	AD/AR	Congenital cataract
				Microcornea
		***GALT*** (p.E201K)	AR	Galactosemia
				Vitreous haemorrhage
				Amblyopia
				Congenital cataract
		*GJA8* (p.L26P)	AD/AR	Congenital cataract
5.	Congenital cataract (no further information)	*CRYAA* (p.R116C)	AD/AR	Congenital cataract
				Microcornea
		***ALDH18A****1* (p.R483H)	AD/AR	Warburg micro syndrome
				Retinopathy
				Microcephaly
				Congenital cataract
6.	Bilateral congenital pulverulent cataract, bilateral aphakia, previous bilateral retinal detachment, glaucoma suspect, naevus, hypertension, high cholesterol, and kidney stones.	*CRYGD* (p.W157*)	AD/AR	Congenital cataract
				Microcornea
		*POMGNT1* (p.D556N)	AR	Muscle–eye–brain disease
				Muscular dystrophy
				Retinitis pigmentosa
				Brain malformations
				Juvenile cataract
7.	Congenital cataract (no further information)	*CRYBA1* (p.G91del)	AD	Congenital cataract
		*CRYAA* (p.C131F)	AD/AR	Congenital cataract
8.	Congenital cataract (no further information)	***CBS*** (p.R224C)	AR	Homocystinuria with ocular complications
				Osteoporosis
				Vascular complications
				Intellectual disability
				Congenital cataract
		***NOD2*** (p.G908R)	AD/AR	Crohn’s disease
9.	Bilateral congenital cataract, high amblyopia and grade 1 spondylolysis in all the family members	*CHMP4B* (E161Q)	AD	Congenital cataract
		***LRP5*** (p.S1260F)	AR	Vitreoretinopathy
				primary open-angle glaucoma
				Osteoporosis–eudoglioma syndrome
				Congenital cataract
10.	Congenital cataract (no further information)	***POMT2*** (p.Y574C)	AR	Walker–Warburg syndrome (cataract, microphthalmia, buphthalmos, and Peters anomaly
11.	Congenital cataract (no further information)	*GJA8* (G22R)	AD/AR	Microcornea
				Congenital cataract
		*GJA8* (V24L)	AD/AR	Microcornea
				Congenital cataract
12.	Congenital cataract (no further information)	*GJA8* (S197F)	AD/AR	Microcornea
				Congenital cataract
13.	Congenital cataract (no further information)	***GALT*** (p.L26P)	AR	Galactosemia
				Vitreous haemorrhage
				Amblyopia
				Congenital cataract

Note: Novel genes are indicated in bold. Autosomal dominant congenital cataract (ADCC), autosomal dominant (AD), autosomal recessive (AR).

## Data Availability

The datasets presented in this article are not readily available due to confidentiality protocols employed by the hospital from which the data is sourced. Requests to access the datasets should be directed to v.berry@ucl.ac.uk.
